# Pretreatment Geriatric Assessments of Elderly Patients with Glioma: Development and Implications

**DOI:** 10.14336/AD.2019.0527

**Published:** 2020-03-09

**Authors:** Yaning Wang, Binghao Zhao, Wanqi Chen, Lei Liu, Wenlin Chen, Lizhou Zhou, Ziren Kong, Congxin Dai, Yu Wang, Wenbin Ma

**Affiliations:** Departments of Neurosurgery, Peking Union Medical College Hospital, Chinese Academy of Medical Sciences and Peking Union Medical College, Beijing, China

**Keywords:** glioma, geriatric, assessment, pretreatment

## Abstract

Glioma is the most frequent primary brain tumor affecting adults, and the most lethal type is glioblastoma (GBM); currently, the available therapies only provide palliation. The treatments for low-grade glioma (LGG) include neurosurgical resection, watchful waiting, radiotherapy and chemotherapy, while the therapeutic strategies for high-grade glioma (HGG), particularly in elderly patients, have evolved to include radiotherapy, chemotherapy, and targeted monotherapy based on the characteristics of the investigated patients. Proper assessments aiming to predict and achieve the most satisfying prognosis among patients prior to surgery, radiotherapy, chemotherapy, targeted therapy or immunotherapy help summarize the pretreatment characteristics of patients, providing doctors comprehensive information to consider while determining whether the patients could benefit from ongoing treatments and deciding the proper treatment strategy for subsequent phases. This article aims to rigorously review the most recent evidence and discuss current mainstream assessments before the initiation of proper treatments for glioma, thus highlighting the potential necessity of pretreatment assessments.

Glioma is the most common intracranial primary malignant tumor in adults, and the most malignant type is glioblastoma (GBM), which has an increasing incidence among the elderly. The incidence in individuals older than 65 years (the threshold age for inclusion in the elderly population according to the NCCN guidelines) is 2.63 times higher than that in younger individuals [1, 2]. Since an advanced age is associated with a poor prognosis and more complicated physical conditions leading to reduced tolerance and decreased efficacy of treatment, optimal treatment strategies must be developed, and broad assessments of patients’ relevant health status must be performed before surgery, chemotherapy, radiotherapy (RT), targeted therapy or even immunotherapy. Proper assessments enable clinicians to provide precise and individual treatments in clinical practice. Notably, glioma has a relatively low incidence among all types of cancers; thus, sufficient high-quality studies or evidence may not be available for some subjects listed in this review. We do not simply constrain the information to glioma and address the significance of pretreatment assessments from the perspective of the common features shared by glioma and other similar cancers. Our study aims to review the most recent high-quality evidence from studies involving geriatric patients with glioma who must decide which type of treatment to undergo to receive the greatest possible benefit, namely, the most beneficial individual therapy and proper assessment strategy.

**Table 1:** Characteristics of Clinical Trials Relating to Treatments (Surgery, Radiotherapy, Chemotherapy and chemoradiotherapy) for Glioma.

First author/published year	Patients’ characteristics (mean age, M/F)	Intervention (number)	Comparison (number)	Results	Follow-up	Study design
Surgery						
Gupta et al/2018 [3]	6.3 (3.3-17.5), 23/27	biopsy	NA	In 50 patients with DIPG going biopsy, 46 successfully captured tissue samples	Followed until death	Single-arm clinical trial
Kellermann et al/2017 [4]	74 (70-87), 129/101	stereotactic biopsy	NA	Two hundred and thirty nine of 230 elderly glioma patients received stereotactic biopsy, 222 achieved histopathologic diagnosis, and 171 received further adjuvant therapy	1 year	Retrospective study
Tanaka et al/ 2013 [5]	74.1 (66-87), 61/44	stereotactic biopsy (52)	surgery (53)	Complications of postoperative bleeding is higher in patients undergoing stereotactic biopsy than in lesion removal surgery	5 year	Retrospective study
Ostrom et al/ 2018[18]	NA (59-64), 56/44	NA	NA	Incidence of glioma and 1-year and 5-year survival rates vary significantly by race and ethnicity with non-Hispanic whites having higher incidence and lower survival rate.	14 year	Retrospective study
Radiotherapy						
Keime et al/2007[40]	73 (70-85), 51/30	radiotherapy plus supportive care (39)	supportive care (42)	Radiotherapy brings better outcome than supportive care in geriatric GBM patients	4 year	RCT
Douw et al/ 2009[41]	44.2, 35/30	NA	NA	Side effect of cognitive loss could only be found in low-grade young glioma patients with RT hindering our decision making	12 year	Prospective clinical study
Malmstrom et al/2012[42]	no less than 60, 118/80	hypofractionated RT (98)	standard RT (100)	OS in geriatric GBM patients receiving hypofractionated RT is comparable in those with standard radiotherapy	9 year	RCT
Roa et al/2004 [43]	elder than 60, 55/40	standard RT (48)	short-course RT (47)	OS in geriatric GBM patients receiving hypofractionated RT is comparable in those with common radiotherapy	5 year	RCT
Roa et al/2015 [44]	no less than 50, 46/52	short-course RT (50)	standard RT (48)	Hypofractionated radiotherapy regimens did not reveal different OS	3 year	RCT
Bent et al/2005[45]	38.8, 191/115	early RT (after surgery) (154)	deferred RT (152)	Early radiotherapy after surgery lengthened PFS but not progression-free survival (PFS)	7.8 year	RCT
Chemotherapy and chemoradiotherapy						
Malmstrom et al/2012 [42]	no less than 60, 173/118	TMZ and hypofractionated RT (191)	standard RT (100)	Two weeks of massive fractionation RT or single TMZ could benefit geriatric GBM patients compared to six-week RT regimen	9 year	RCT
Stupp et al/2009[56]	360/213	RT with concomitant adjuvant TMZ (287)	RT (286)	Six cycles of TMZ adjuvant chemoradiotherapy prolongs the survival of elderly patients with GBM	2 year	RCT
Perry JR et al/2017[57]	73 (65- 90), 43/219	Short course RT (40Gy/15F) with concomitant adjuvant TMZ (281)	Short course RT (281)	The addition of temozolomide to short-course radiotherapy resulted in longer survival than short-course radiotherapy alone	Almost all followed until death	RCT

Abbreviations: DIPG, diffuse intrinsic pontine glioma; OS, overall survival; PFS, progression-free survival; GBM, glioblastoma; RT, radiotherapy; TMZ, temozolomide; RCT, randomized controlled trial; NA, not available

## Surgery Among Geriatric Patients with Glioma

The appropriate surgical methods for geriatric patients with glioma remain controversial. Studies involving surgery are summarized in Table 1 [3-5,18]. According to the 2017 National Comprehensive Cancer Network (NCCN) Clinical Practice Guidelines in Oncology focusing on older adult oncology, patients with GBM aged greater than 70 years achieve better prognoses when their tumor lesions are completely removed than when they are partially removed. The results obtained in younger patients are similar to those obtained in elderly patients (≥70 years) [6, 7]. A key question is whether the different prognoses originate from the operative methods (total or partial removal) or differences in the participants enrolled in the trials. The use of multiple bits and single bits in an “open biopsy” through a craniotomy resection (surgical) or endoscopic procedure leads to histological or cytological diagnoses similar to those in a stereotactic biopsy. However, some indicators, such as intraoperative bleeding, the operation time, postoperative complications, postoperative bleeding, cognitive function loss, hospital stay, recovery time, etc., may be highly variable [8].

“Stereotactic biopsy”, which is also known as stereotactically directed biopsy, was among the first minimally invasive procedures used in neurosurgical management. Due to advances in neuroimaging devices, numerous minimally invasive lesions are diagnosed at an increasing rate. Additionally, intraoperative magnetic resonance imaging (MRI) has transformed a blind procedure into a visual procedure. As geriatric patients (≥70 years) may not successfully survive the arranged surgeries, approximately 20% of these patients may have unexpected complications; therefore, a stereotactic biopsy is a good choice. Currently, stereotactic biopsy provides a rapid histological or cytological diagnosis with a secure profile, thus establishing its wide use in the field of neurosurgery to obtain an accurate diagnosis and manage the treatment of the central nervous system.

Gupta*et al*. [3] reported data from 50 patients with diffuse intrinsic pontine glioma (DIPG) who underwent open biopsy. Among these patients, tissue samples were successfully removed from 46 patients, assisting the researchers in making conclusive diagnoses and further treatment decisions. After the initial diagnosis, a biopsy is regarded as a safe strategy in geriatric patients with glioma to guide subsequent treatment with a low operative risk. In another retrospective study [4], 229 of 230 elderly patients with glioma underwent stereotactic biopsy, including 222 patients who obtained a histopathologic diagnosis and 171 patients who received further adjuvant therapy. In summary, similar to a single biopsy, stereotactic biopsy is a reliable, safe surgical option and an optional preassessment method before relevant adjuvant therapy is performed. Stereotactic biopsy requires a smaller incision than open biopsy, which increases the uncertainty of patients’ intracranial conditions; thus, surgeons are unable to determine the complete situation during stereotactic biopsy. Intraoperative bleeding in stereotactic biopsy appears to occur less frequently than that in open biopsy. In addition, regarding complications after surgery, such as postoperative bleeding, the risk of intracranial hemorrhage in patients undergoing stereotactic biopsy is significantly higher than that in patients undergoing standard lesion removal in an open biopsy [5]. Overall, the prognoses of elderly patients with glioma worsen with increasing age, and surgery for tumor lesions is still recommended as a relatively good strategy despite several acknowledged drawbacks and potential risks.

### Preoperative Assessments of Geriatric Patients with Glioma

Preoperative assessments of elderly patients with glioma include assessments of cognitive function, complications, frailty, activities of daily living, quality of life, nutritional status, laboratory parameters and geriatric syndromes, and these assessments are of paramount importance when making treatment decisions and can assist in providing prognostic information [9]. To the best of our knowledge, age and the extent of resection (EOR) are two of the strongest prognostic factors in GBM; the prognosis and survival time of patients with glioma also vary by race and ethnicity [10-18]. The studies describing preoperative assessments are summarized in Table 2 [10-17].

Cognitive function is an essential component of all preoperative assessments and is deemed an independent prognostic factor among geriatric patients with glioma who are undergoing surgery and corresponding RT. Derek*et al*. and Johnson*et al*. [10] conducted a series of cognitive functional tests in 91 patients who were newly diagnosed with glioma and had undergone surgery without further treatment. The prognosis after surgery was worse among the elderly patients with glioma and was specifically related to poor communication and behavioral functions. Cognitive attention was also associated with unsatisfactory prognoses (all P-values <0.01). Notably, we could predict the postoperative prognoses of these patients based on the results of preoperative cognitive functional assessments.

The Mini-Mental State Examination (MMSE) [19] and Montreal Cognitive Assessment (MoCA) [20] are commonly used to assess patients’ cognitive function in clinical practice. Recently, Rambeau*et al*.[11] performed a large-scale prospective cohort study to compare the effectiveness of the MMSE and MoCA in evaluating patients’ cognitive function and revealed that the MoCA was superior in assessing patients’ cognitive function because the MoCA identified more impairments in cognitive function than the MMSE.

**Table 2:** Preoperative, Preradiotherapy, Prechemotherapy Assessments for Glioma and Other Cancer Patients.

First author/published year	Patients' characteristics (mean age, M/F)	Intervention (number)	Comparison (number)	Results	Follow-up	Study design	Assessment items
Preoperative assessments							
Johnson et al/2012[10]	53.9, 55/36	WAIS-R, WAIS-III, HVLT-R questionnaire	NA	Executive function and attention are cognitive domains related to prognosis of GBM	9 year	retrospective study	cognitive function
Rambeau et al/2018 [11]	78 (70-93), 25/41	MMSE (13)	MoCA (44)	MoCA seems to be more relevant to screen cognitive impairment	1 year	RCT	cognitive function
Fiorentino et al/2012 [12]	72 (65-81), 17/18	RTCHT (35)	NA	Comorbidity assessments is an appropriate tool for predicting prognosis of elderly patients with GBM	6 year	single-arm clinical trial	comorbidity
Ening et al/2015 [13]	62 (15-84), 117/116	NA	NA	Besides old age and low KPS score, higher CCI is significantly associated with poor OS and PFS in dismal GBM, hence decides patient stratification	5 year	retrospective review	comorbidity
Cloney et al/2015 [14]	no less than 65, NA	surgical resection	biopsy	Frailer elderly GBM patients are less likely to undergo surgical resection, have longer hospital stay, more complications, and less OS	12 year	retrospective study	frailty
Peters et al/2014 [15]	mean 50, 161/76	NA	NA	Greater degree of fatigue was associated with poor survival in recurrent HGG patients, which shows fatigue is an independent predicator for OS rather than QoL	2 year	prospective cohort study	fatigue and QoL
Borg et al/2011 [16]	mean 60.1, 419/266	NA	NA	GBM patients with pre-operative hypoalbuminaemia status have less post-operative OS than normal albumin	10 year	retrospective study	lab indicators
He et al/2017 [17]	44 (5-78), 197/129	surgery (some received aggressive adjuvant treatment)	NA	Newly diagnosed HGG patients with elevated fibrinogen level and decreased albumin levels have more risk of tumor progression and death	2 year	single-arm clinical trial	lab indicators
Preradiotherapy assessments							
VanderWalde et al/2017 [49]	72.5 (65-92), 24/22	NA	NA	Cancer patients failed to receive pre-treatment are more likely to have low health-related quality of life.	2 year	prospective cohort study	health-related quality of life
Antonio et al/2018 [50]	79.5 (75-87), 76/9	NA	NA	Fit NSCLC patients have longer mOS than medium-fit; patients with higher VES-13 will have shorter mOS and high risk of G3-4 toxicity	8 year	prospective cohort study	geriatric assessments
Pottel et al/2014 [51]	72 (65-86), 86/14	NA	NA	CGA helps to identify the evolution of health problems and is indicative of quality of life in patients with head and neck carcinoma	2 year	prospective cohort study	geriatric assessments
Gielda et al/2011 [52]	64 (30-84), 22/32	NA	NA	Weight decrease strongly correlates with declined OS/PFS of NSCLC patients. Weight change during treatment should be treated as potential predicator	11 year	prospective cohort study	nutritional status
Fiorentino et al/2012 [12]	more than 65, 17/18	NA	NA	Elderly GBM patients with lower CCI score will have a longer OS than those with higher	6 year	prospective cohort study	comorbidity
Chaichana et al/2011 [53]	older than 65, 67/66	NA	NA	KPS score less than 80, motor deficit, language deficit, cognitive deficit are independently associated with decreased OS of elderly GBM patients	10 year	retrospective study	KPS score, motor, language cognitive function
Prechemotherapy assessments							
Wick et al/2017[47]	57.7 (21.2-82.3), 265/172	lomustine plus bevacizumab (288)	lomustine alone (149)	Despite somewhat prolonged PFS, lomustine plus bevacizumab did not confer a survival advantage over treatment with lomustine alone in glioblastoma patients. The combined therapy affected neither health related quality of life nor neurocognitive function. The MGMT status is prognostic	3 year	RCT	cognitive functiion
Aparicio et al/2013[59]	80 (75-91), 66/57	fluorouracil (62)	fluorouracil with irinotecan (61)	Cognitive function and autonomy impairment assessed by MMSE, IADL, MMSE are predictive of severe toxicity or unexpected hospitalization of elderly metastatic colorectal cancer patients	7 year	RCT	cognitive function, autonomy impairment
Aaldriks et al/2011[60]	77 (71-92), 90/112	NA	NA	Inferior MNA and MMSE scores increase the probability of elderly cancer patients not to complete hemotherapy	3 year	prospective cohort study	MNA, MMSE

Abbreviations: GBM, glioblastoma; WAIS-R, wechsler Adult Intelligence Scale-revised; WISC-III, Wechsler Intelligence Scale for Children-III; HVLT-R:Hopkins Verbal Learning Test-Revised; RCT, randomized controlled trial; MMSE, Mini-mental State Examination; MoCA, Montreal Cognitive Assessment; RTCHT, radiochemotherapy; CCI, Charlson comorbidities index; KPS, Karnofsky score; MGMT, O6-methylguanine-DNA methyltransferase; mOS, median overall survival; PFS, progression-free survival; HGG, high grade glioma; QoL, quality of life; NSCLC, non-small cell lung cancer; VES-13, The Vulnerable Elderly Survey 13; G3-4, grade 3-4; IADL, Instrumental Activities of Daily Living Scale; NMA, Mini Nutritional Assessment; NA, not available.

Proper comorbidity assessments can help clinicians evaluate the overall physical conditions of older patients because of their poor tolerance for surgeries compared to adults. Fiorentino*et al*. [21] used the Charlson Comor-bidity Index (CCI) to evaluate the comorbidities of elderly patients with GBM. Thirty-three participants were enrolled in their study, and the patients with the lower CCI scores had significantly longer overall survival (OS). Similarly, in a retrospective study involving 233 patients who were newly diagnosed with GBM, Ening*et al*. [13] investigated the associations between patients’ OS and baseline characteristics, clinical treatment conditions and comorbidities. Poor OS and progression-free survival (PFS) were significantly correlated with an older age (>65 years), low Karnofsky performance scores (KPSs) (≤70) or a high CCI (>3). Based on the abovementioned studies, the CCI is a useful tool to comprehensively and effectively evaluate the comorbidities of patients with glioma, particularly elderly patients with GBM.

Assessments of frailty in elderly patients are deemed an essential component of the Comprehensive Geriatric Assessment (CGA). Michael Cloney*et al*.[14] further investigated the status of frailty in elderly patients, particularly elderly patients with GBM. In their retrospective study, the researchers found that severely frail individuals were less likely to have their lesions completely removed (P=0.02), had a relatively longer hospital stay (P=0.0061), had a higher risk of developing surgical complications (P=0.0123) and had a shorter OS (P=0.0028). According to previously reported evidence, a considerable number of neurosurgeons optimize their clinical decision-making processes for intended surgical candidates based on the results of frailty status assessments and cognitive function tests. The abovementioned studies addressed the significant role of geriatric assessments in determining the frailty status of geriatric patients with glioma. The FRAIL SCALE [22] is recommended as a standard method for evaluating the frailty status.

Currently, the quality of life is becoming a popular indicator of the conditions associated with prognosis and used to predict patient prognosis. In a prospective study involving 237 patients with recurrent high-grade glioma (HGG), Katherine B. Peters*et al*. [15] aimed to investigate the effects of the quality of life and frailty status on the prognosis of these patients using corresponding questionnaires. According to a strict statistical analysis, the quality of life was not an independent predictor of the prognosis of patients with HGG. However, patients with glioma who recorded low quality of life (QLQ) scores were likely to suffer from communication difficulties, logic disorders and a loss of motor function, which are imperative factors in predicting prognosis. Clinical practitioners have always adopted the Quality of Life Questionnaire C30 (QLQ-C30) [23] to assess the quality of life in patients with cancer. The Quality of Life Questionnaire BN20 (QLQ-BN20) has been used in combination with the QLQ-C30 in patients with glioma. The Functional Assessment of Cancer Therapy-Brain (FACT-Br) questionnaire is widely used in patients with brain cancer and mainly aims to assess the QOL according to general, social/family, emotional and functional brain cancer-specific issues, including concentration, memory, seizures, eyesight, hearing, speech, personality, expression of thoughts, weakness, coordination, and headache [24]. Both the QLQ-BN20 and FACT-Br are regarded as valid and reliable tools in populations with primary brain cancer. When applying these two tools, we must balance the strengths and weaknesses of each item. Another tool, i.e., the Short Form Health Survey-36 (SF-36), is a 36-item, patient-reported survey of patient health. The SF-36 contains eight 0-100-point scales with equal weights. The eight scales include vitality, physical functioning, bodily pain, general health perceptions, physical functioning, emotional functioning, social functioning, and mental health and are commonly used to assess the health status of individuals. Despite its wide use in health status evaluations, the SF-36 has limitations as the survey does not consider a sleep variable and has a low response rate in elderly patients [25, 26]. The SF-36 should be used cautiously based on these limitations.

Hemoglobin concentrations and serum albumin levels have been used to conveniently and accurately assess the nutritional status of patients with glioma. The serum albumin level is an important indicator of the systemic inflammatory response and nutritional status of patients and indicates the prognosis of patients with bladder cancer, ovarian cancer, soft tissue sarcoma, breast cancer, etc. [27-30]. According to Borg and Schwartzbaum*et al*. [16, 31], low serum albumin levels are significantly correlated with a shorter OS.

Fibrinogen is a key regulator of inflammation and cancer progression that has important functions in tumor cell proliferation, migration and angiogenesis [32]. Higher serum fibrinogen levels predict a poor OS in patients with rectal cancer, non-small cell lung cancer (NSCLC), renal cell carcinoma, breast cancer, *etc*. [33-35]. In 2015, Matsuda*et al*. [36] first constructed the fibrinogen albumin (FA) score, representing a new assessment system for predicting the prognosis of patients with esophageal carcinoma based on the preoperative fibrinogen and albumin levels. He*et al*. [17] further analyzed the FA scores of 326 patients who were newly diagnosed with HGG and found that patients with HGG with higher FA scores exhibited poorer OS and PFS. The risk of tumor progression was 4 times greater in the patients with high fibrinogen and low albumin levels, and the risk of mortality was 4.23 times greater in such patients. Multivariate confounder analyses have shown that FA is an independent predictor of OS and PFS.

Recently, increasing studies [37, 38] have focused on the relationship between inflammatory factors and prognoses in patients with malignant tumors, and inflammatory factors have been included in several scales used for cancer prognosis assessments. Inflammatory factors, such as C-reactive protein and serum albumin, are also reliable and sensitive indicators of the acute response of tumor cells to proper interventions [39]. Although studies investigating laboratory biomarkers in elderly patients with GBM are still limited, routine assessments of the levels of hemoglobin, albumin, fibrinogen, and other inflammatory factors are recommended before surgery. Age and EOR are strongly predictive of the GBM prognosis as younger patients with a smaller EOR exhibit more satisfying prognoses than older patients with a larger EOR. Age, sex, race and ethnicity influence the incidence and survival time of glioma. For example, the incidence risk in non-Hispanic whites is twice as high as that in blacks and 25% higher than that in Hispanic whites; the prognosis also varies according to race and ethnicity. In addition to these well-known factors, repeated procedures and surgeries are confirmed to improve patients’ OS. The GBM subtype was confirmed to affect this parameter in a univariate analysis but not a Cox model [18, 40].

### RT in Geriatric Patients with Glioma

A precise standard for the treatment of GBM in elderly patients does not exist. The optimal RT regimen in these patients remains to be established. According to the 2018 NCCN guidelines for older adult oncology, the decision to offer RT to older patients should be individualized based on several principles, including an evaluation of the benefits and risks associated with RT, consideration of the patient’s status, and an understanding of the nature of the disease. The studies analyzing RT are summarized in Table 1 [40-45].

According to the results of the ANOCEF trial, RT leads to better outcomes than supportive care in elderly patients with GBM without reducing the quality of life or cognition[40]. Since evidence of cognitive side effects is available only for nonelderly patients with low-grade tumors, the risk of the loss of cognitive function with RT should not be included in the decision-making process regarding the treatment of elderly patients [41].

Several studies have reported the noninferiority of shorter hypofractionated RT protocols. Malmström*et al*. [42] and Roa*et al*. [43] performed prospective studies to improve the treatment of elderly patients and curtail lengthy RT treatments and showed that no difference in OS existed between elderly patients with GBM who received hypofractionated RT and patients receiving the commonly used RT. Regarding the reduced treatment time, a short-course RT regimen may be recommended as an attractive treatment option to improve treatment tolerability, particularly in frail patients. Currently, hypofractionated RT regimens include 40 Gy/15 F, 34 Gy/10 F, and 25 Gy/5 F, which do not yield significant differences in OS [44].

The onset of RT was studied in a prospective, randomized trial conducted by Van Den Bent*et al*., who assessed early and delayed RT in patients with low-grade glioma, astrocytoma and oligodendroglioma. Early RT after surgery lengthened the PFS but did not impact the OS [45]. The RT options available to elderly patients with a good status, low-grade gliomas, isocitrate dehydro-genase (IDH) mutations, epidermal growth factor receptor (EGFR) amplification, 1p/19q deficiencies or O6-methylguanine-DNA methyltransferase (MGMT) promoter methylation considerably vary if these patients are carefully monitored[46, 47]. In addition to the aforementioned molecular markers, the statuses of other targeted genes, distinct biological processes, and mutations may be distinguished, and thus, the best RT alternative has not been verified [47]. Medical practitioners must maximize the benefits of individualized RT based on the patients’ characteristics.

### Pre-RT Assessments of Geriatric Patients with Glioma

Pre-RT assessments are designed to explore the relationship between the indicators before RT and the prognosis of the treatment. Pre-RT assessments of patients with central nervous system tumors, particularly gliomas, are currently being investigated based on known research directions, including age, fatigue, comorbidities, cognitive abilities and a new screening approach, i.e., CGA.

RT is well tolerated, and thus, age alone should not be a limiting factor in elderly patients with cancer[48]. Therefore, screening elderly patients who might benefit from RT using scientific assessment tools may be necessary to deliver quality cancer care. The studies performing pre-RT assessments are summarized in Table 2 [12, 49-53].

The CGA is a multidimensional, interdisciplinary approach aiming to assess patients’ general health status, including functional, cognitive, nutritional, social, medical and psychological parameters, and is considered the most efficient method for screening the functional status and reserve capabilities of elderly patients [54]. The feasibility of using the CGA as a predictor of the quality of life and outcomes in elderly patients with cancer receiving RT has been reported in a prospective study conducted by Vander Walde*et al*., who found that a lower instrumental activities of daily living (IADL) score was associated with a continued decline and lack of recovery of health-related quality of life (HRQoL) [49]. Another prospective study showing the prognostic role of CGA was conducted by Antonio*et al*., who included 85 patients aged ≥75 years with locally advanced NSCLC [50]. Additionally, Pottel*et al*. showed that the CGA was able to identify the evolution of multidimensional health problems and indicated the quality of life in elderly patients with head and neck carcinoma who were undergoing curative RT [51].

Nutritional deficiency is a common but serious condition that is underdiagnosed in elderly patients with cancer. Jager-Wittenaar*et al*. reported that the prevalence of malnutrition in patients treated for oral or oropharyngeal cancer is 16% [55]. A poor nutritional status could increase symptoms of radiation toxicity, such as dermatitis, sore throat, fatigue and anorexia. The strong correlation between weight changes during conformal RT (CRT) and OS/PFS reported by Gielda*et al*. suggests that the nutritional status might be useful as a complementary source of predictive information [52]. According to the 2018 NCCN guidelines for older adult oncology, nutritional support is recommended for patients receiving RT. The malnutrition screening tools commonly used in clinical practice include the body mass index (BMI), Mini Nutritional Assessment (MNA) scale score, etc.

Regarding fatigue, Peters*et al*. reviewed the records of 237 patients with recurrent HGG and found that fatigue was a strong independent predictor of survival [15].

Comorbidities are also associated with the prognosis after RT. Thirty-five patients older than 65 years with a KPS score greater than 60 and histological proof of GBM were treated at the same center and evaluated retrospectively. All patients underwent complete or partial excisions or biopsy, followed by chemoradio-therapy and adjuvant temozolomide (TMZ) therapy. The patients with low CCI scores experienced longer survival times than the patients with higher CCI scores [12].

Chaichana*et al*. [53] performed a retrospective analysis of 129 patients older than 65 years who experienced nonbiopsy removal of intracranial GBM at the same institution after controlling for perioperative and postoperative factors associated with outcomes. By the final follow-up, all patients had died, with a median survival of 7.9 months. The preoperative factors that were independently associated with reduced survival included a KPS<80 (P=0.001), chronic obstructive pulmonary disease (P=0.01), dyskinesia (P=0.01), speech impairment (P=0.005), recognition dysfunction (P=0.02), and tumor size>4 cm (P=0.002).

All aforementioned studies have shown that a variety of factors might affect the tolerance of elderly patients with glioma to RT. Hence, clinicians should conduct a comprehensive evaluation of the corresponding factors to design a more suitable treatment plan for elderly patients before deciding to administer RT, allowing the patient to obtain more benefits. However, current research is still limited, and high-level evidence is lacking. Additional studies are needed.

### Chemotherapy and Chemoradiotherapy for Geriatric Patients with Glioma

When considering chemotherapy and chemotherapy protocols, the 2017 NCCN guidelines for geriatric oncology patients suggest that treatment with TMZ alone is no less effective than RT alone in elderly patients with astrocytoma and GBM (>64 years). The studies analyzing chemotherapy are summarized in Table 1 [42, 56, 57]. Compared with RT, the use of TMZ alone can prolong event-free survival in patients with MGMT promoter methylation [58]. In patients with GBM aged greater than 70 years, two weeks of massive fractionation RT or a single treatment with TMZ improved patient survival more than a standard six-week RT regimen. Furthermore, the study confirmed that the methylation status of the MGMT promoter played a guiding role in the use of TMZ [42]. Concurrent chemoradiotherapy and 6-course TMZ adjuvant chemotherapy have been shown to prolong survival in elderly patients with GBM aged 60-70 years[56]. A recent phase 3 trial involving 562 patients aged ≥65 years tested a new chemoradiation strategy in which TMZ was added to a shorter course of RT (40 Gy/15 F). The addition of TMZ to short-course radiotherapy was associated with a significant survival benefit over short-course radiotherapy alone among elderly patients with newly diagnosed glioblastoma, which was particularly evident in patients with a methylated MGMT status [57].

### Prechemotherapy Assessments of Geriatric Patients with Glioma

Chemotherapy and prechemotherapy assessments have been conducted in elderly patients with cancer in many studies, which are summarized in Table 2 [47, 59, 60]. The results of these studies confirmed that general abilities in daily life, complications, the nutritional status, cognitive abilities, the psychosomatic state and frailty are related to possible toxic reactions during or after chemotherapy and the patient’s tolerance to chemotherapy [61].

Thomas Aparicio*et al*. [59] conducted a prospective study involving elderly patients with metastatic gastrointestinal tumors. This study involved 123 patients aged on average 80 years, and their complications, cognition and daily living abilities were evaluated before chemotherapy. The results of this study showed that cognitive impairment (MMSE≤27 / 30; odds ratio (OR), 3.84) and poor daily living ability (OR, 4.67) were associated with chemotherapy-related grade 3-4 side effects in elderly patients with cancer. Based on this finding, these types of abilities must be evaluated before administering chemotherapy.

Another prospective study was conducted by Arti Hurria*et al*. [62]. This study analyzed 500 elderly patients with cancer aged 65 years or older (including patients with gastrointestinal cancer, breast cancer, urogenital carcinoma, etc.) recruited from seven medical centers. The treatment plans, laboratory investigations and geriatric assessments, including evaluations of cognitive function, complications, the psychological status, the nutritional status, etc., were evaluated. In addition, the occurrence of chemotherapy-related toxic reactions was also analyzed. Notably, 53% of the patients developed grade 3-5 chemotherapy-related side effects after treatment. Eleven risk factors, including complications (creatinine clearance rate<34 ml/min), were identified. No significant correlations were observed among all parameters, such as frailty (restricted walking for 1 block) or mental state.

During the same period, A. Aaldriks*et al*. [60] performed a comprehensive CGA before and after chemotherapy in elderly patients with cancer. The authors analyzed the results of various assessment items and patient mortality after chemotherapy. The nutritional status and cognitive function scores after chemotherapy were significantly worse than those before chemotherapy (P=0.001 and P=0.04, respectively). The patients with a low nutritional index score (MNA score) and a high frailty index score (Groningen Frailty Index, GFI) exhibited significantly higher mortality after chemotherapy. Fifty-one patients were re-evaluated after chemotherapy, and chemotherapy significantly reduced the cognitive function score (MMSE score) but had a minimal effect on the frailty index (GFI).

Regarding psychosis, a prospective study involving 83 elderly patients with ovarian cancer whose average age was 76 years was conducted by G. Freyer [63]. The researchers evaluated comorbidities, cognitive functioning, the nutritional status, mental and emotional states (i.e., whether they were associated with anxiety or depression) and drug delivery protocols. Depression (P=0.006), a poor general condition (P=0.026) and limited ability to live independently (P=0.048) were independent risk factors for severe chemotherapy-induced toxicity. Depression (P=0.003) and drug use (more than 6 types of drugs per day, P=0.043) were independent predictors of the OS.

Harvey Jay Cohen*et al*. [64] conducted a study investigating frailty in elderly patients with cancer aged 65 years or older. Five hundred patients were assessed for frailty before chemotherapy and divided into “robust/nonweak” (50%), “prefrail” (39%) and “frail” (11%) groups. The degree of frailty was correlated with the patients’ prognosis and chemotherapy-induced toxicity. These results highlight the importance of evaluating patients’ frailty before administering chemotherapy.

Prechemotherapeutic assessments have been widely performed in elderly patients with cancer. Alba Fiorentino*et al*. [21] studied the effects of comorbidities on the tolerance of elderly patients with GBM to RT and TMZ chemotherapy. Thirty-five patients with GBM older than 65 years were investigated, and all patients were treated with surgery, concurrent chemoradiotherapy and adjuvant TMZ chemotherapy. The patients with low complication index scores (CCI, <3) experienced a longer OS (22 months versus 10 months), but statistical evidence showing that comorbidity was a predictor of PFS was not found.

Based on the significant differences in tolerance to treatments between elderly patients and adult patients, pretreatment assessments are particularly important. All studies mentioned above reported a relationship between prechemotherapeutic assessments and tolerance to chemotherapy in elderly patients with cancer, thus confirming the importance of such evaluations. Clinicians should fully use these noninvasive approaches to provide safer and more effective treatments to elderly patients.

### Immunotherapy Assessments in Geriatric Patients with Glioma

Immunotherapy intends to stimulate patients’ immune systems and promote an immune-mediated anti-tumor response. This interesting topic has recently provided new therapeutic strategies for glioma.

Antibody-dependent cellular cytotoxicity (ADCC), cancer immunization, oncolytic viruses, chimeric antigen receptor T-cell (CAR-T) therapy, cytokine treatment, dendritic cell therapy, and checkpoint blockade all contribute to immunotherapy. The effective profiles of immunotherapy have been validated in patients with some other cancers, such as lung and breast tumors. Antibodies against HER2/neu and tumor-specific gangliosides are currently investigated for ADCC in patients with glioma [65, 66], and IL-2 therapy produced limited benefits [67]. A phase III trial investigating DCVax-L, which is an autologous tumor lysate-pulsed DC vaccine, has shown significantly prolonged mOS in patients with GBM[68]. CAR-T-mediated therapies targeting GBM markers, such as EGFRvIII, IL-13Rα2, HER2, and EphA2, are also being tested in ongoing clinical trials (NCT01454596, NCT02664363, NCT02209376, NCT 02208362, NCT02442297, NCT01109095, and NCT02575261).

Convincing real-world data regarding glioma remain insufficient or inconclusive, particularly in elderly patients, and few studies have performed preimmuno-therapy assessments. The existing studies mainly focus on imaging evaluations, such as perfusion-weighted imaging (PWI), diffusion MRI, etc. Currently, the definitions of PFS and the clinical benefits of immunotherapy in patients with glioma are not well established. These radiological markers are reliable tools used to help assessments of the potential benefits of immunotherapy and distinguish real tumor progression from pseudoprogression to avoid misdiagnoses [69].

### Geriatric Syndrome and Caregivers

Geriatric syndrome refers to a syndrome characterized by the same clinical manifestations or problems caused by diseases or other causes, including falls, dementia, urinary incontinence, delirium, syncope, depression, pain, insomnia, drug abuse and geriatric Parkinson’s syndrome. Each component of geriatric syndrome can be specifically evaluated. For example, geriatric delirium is a type of acute ambiguous consciousness manifested as a loss of attention, feeling, mental health, and memory as well as psychomotor and sleep problems [70]. The identification of the cause of geriatric delirium and the administration of proper treatments are necessary; actually, geriatric delirium can resolve in a few days or a few months. When pathological lesions are present in patients’ brains or cerebral vessels, delirium may be a risk factor for unsatisfactory prognoses. Assessments of geriatric delirium mainly involve the following aspects: 1. a physical examination of the central nervous system to determine whether substantial pathological lesions are present in the patient’s brain; 2. an evaluation of disruptions in the patient’s vision or hearing; 3. assessments of infections, electrolyte disorders or other types of illnesses; 4. assessments of endocrine function to determine the hormone levels; and 5. evaluations of psychological stress and other social factors [71].

**Table 3:** Summary of Geriatric Syndrome and Caregiver.

First author/published year	Patients’ characteristics (mean age, M/F)	Measurement/method	Results	Follow-up	Study design	Assessment items
Kane et al/2012 [71]	NA	eight geriatric syndromes (multiple comorbidities, cognitive impairment, frailty, disability, malnutrition, impaired homeostasis and chronic inflammation) and survival	Geriatric syndrome helps to understand the survival for younger old persons but provides little information for the very old	NA	systematic review	geriatric assessments
Svendsboe et al/2016 [73]	74.9, 72/114	caregiver burden (RSS), cognitive function (MMSE, CDR-SOB), Neuropsychiatric symptoms (NPI, MADRS, UPDRS), other variables (CIRS, RDRS-2)	Caregivers to people with AD and to people with DLB experience moderate or high caregiver burden with increased risk of psychiatric disorder in early stage of dementia	NA	cross-sectional study	caregiver

Abbrevations: RSS, Relative Stress Scale; MMSE, Mini Mental State Examination; CDR-SOB, Clinical Dementia Rating scale sum of boxes; NPI, Neuropsychiatric In- inventory; MADRS, Montgomery and Asberg Depression Rating Scale; UPDRS, The Unified Parkinson’s Disease Rating Scale; CIRS, Cumulative Illness Rating Scale; RDRS-2, the Rapid Disability Rating Scale-2; AD, Alzheimer’s disease; DLB, dementia with Lewy bodies; NA, not available

Caregivers are also crucial contributors to the process of managing geriatric patients with cancer. Caregivers help send patients to proper medical institutions on time, cope with emergencies, offer daily healthcare, decide the best treatment strategies, address complications, fill the gaps between medical workers and the patient’s family members, promote communication among the patient’s family members, resolve conflicts between patients and doctors and show empathy to special patients [72, 73].

Excellent caregivers act as representatives not only of the patients but also of the patients’ family members in addressing family conflicts or conflicts in medical administration. Proper, effective care also impacts the choice of the best treatment by improving compliance and decreasing the risk of severe complications. When elderly patients with cancer are unable to independently perform essential daily activities, their healthcare, quality of life and even normal life all likely depend on their caregivers[74, 75]. In summary, patient prognoses after relevant therapies depend on their caregivers’ choices. The selection of an ideal caregiver for geriatric patients may improve their living conditions and determine their prognoses. The studies investigating geriatric syndromes and caregivers are summarized in Table 3 [71, 73].

Overall, geriatric patients with GBM represent a very heterogeneous group with epidemiology and management that differ from those in other patient groups. Survival is further limited in elderly patients who are often unable to tolerate multimodal therapy. Because an increasing number of patients are diagnosed with GBM and the aging population is increasing, comprehensive assessments of geriatric patients with glioma must be performed before all types of therapies are administered. Combined with current evidence-based medicine, complete assessments of geriatric patients with glioma, including evaluations of cognitive function, comorbidity, frailty, quality of life, nutritional status, laboratory indicators, etc., can assist clinicians in creating suitable, reasonable, and precise treatment strategies. In addition, these assessments should be performed by neurosurgeons worldwide. Patients with brain cancer primarily present to neurosurgery clinics for the removal of brain tumors using surgical procedures. Neurosurgeons conduct detailed examinations and adequate preoperative preparations (if they decide to perform surgeries) to ensure that the surgery is a safe process. These doctors also conduct pretreatment assessments to predict the prognoses and benefits of their patients through the assigned treatments; therefore, these doctors know whether the ongoing treatment produces a sufficient benefit and are able to identify proper alternative therapeutic strategies if the ongoing treatment is not the best choice. Furthermore, specific assessments of the presence of geriatric syndrome and the availability of caregivers could aid physicians in determining the balance between the potential benefits and risks of each oncological strategy for geriatric patients with GBM.
